# The bioinductive collagen implant yields positive histological, clinical and MRI outcomes in the management of rotator cuff tears: A systematic review

**DOI:** 10.1002/ksa.12429

**Published:** 2024-09-05

**Authors:** Umile Giuseppe Longo, Martina Marino, Alessandro de Sire, Miguel A. Ruiz‐Iban, Pieter D'Hooghe

**Affiliations:** ^1^ Fondazione Policlinico Universitario Campus Bio‐Medico Roma Italy; ^2^ Research Unit of Orthopaedic and Trauma Surgery, Department of Medicine and Surgery Università Campus Bio‐Medico di Roma Roma Italy; ^3^ Physical Medicine and Rehabilitation Unit, Department of Medical and Surgical Sciences University of Catanzaro “Magna Graecia” Catanzaro Italy; ^4^ Shoudler and Elbow Unit. Hospital Universitario Ramón y Cajal Madrid Spain; ^5^ Aspetar Orthopedic and Sports Medicine Hospital Doha Qatar

**Keywords:** bio‐inductive collagen implant, bio‐inductive collagen patch, rotator cuff tears, tendon healing, tissue augmentation, tissue regeneration

## Abstract

**Purpose:**

The aim of this study is to report and discuss the outcomes of clinical, histological and animal studies exploring the application of bio‐inductive collagen implants (BCIs) to partial and full‐thickness rotator cuff tears (PT‐ and FT‐RCTs) in addition to reporting on cost‐related factors.

**Methods:**

Review of literature was performed using the PRISMA guidelines. A systematic electronic literature search was conducted using the CENTRAL, CINAHL, Cochrane Library, EBSCOhost, EMBASE and Google Scholar bibliographic databases. Microsoft Excel was used to create tables onto which extracted data were recorded. Tables were organized based on the research statement formulated using the PICO approach. No statistical analysis was performed.

**Results:**

Nine studies evaluated clinical and MRI outcomes of BCI augmentation for FT‐RCTs, seven evaluated similar outcomes when applied to PT‐RCTs, two additional studies were case reports and three studies assessed application to FT‐ and PT‐RCTs without stratification of results, one of which also reported on histological data. Two studies reported on histological data alone, and finally, two reported on healthcare costs. BCI augmentation, alone and combined with rotator cuff repair (RCR), displays generally good histological, postoperative clinical and MRI outcomes for PT‐ and FT‐RCT treatment. Recent economic analyses seem to be in favour of the use of this procedure, when selected and applied for appropriate patient populations.

**Conclusion:**

Several studies have shown promising results of BCI application to PT‐ and FT‐RCTs, both concomitantly and independently from RCR. Investigations report promising histological characteristics, improved clinical outcomes, increased tendon thickness, reduced defect size and lower re‐tear rates.

**Level of Evidence:**

Level IV.

**Abbreviations**
ABD: abduction
ASES: American Shoulder and Elbow Surgeons
BCI: bio‐inductive collagen implant
ER: external rotation
FE: forward elevation
FF: forward flexion
FT‐RCT: full thickness rotator cuff tear
IR: internal rotation
MCS: mental component score
MRI: magnetic resonance imaging
PCS: physical component score
PT‐RCT: partial thickness rotator cuff tear
RC: rotator cuff
RCR: rotator cuff repair
RCT: randomized controlled trial
ROM: range of motion
RSA: reverse shoulder arthroplasty
SANE: single assessment numeric evaluation
SEM: standard error of mean
SST: simple shoulder test
VR‐12: veterans RAND 12‐item health survey
WORC: Western Ontario Rotator Cuff Index

## INTRODUCTION

Rotator cuff (RC) tears have an incidence between 16% and 34% in the general population and are considered a debilitating condition that may lead to pain, disability and psychosocial dysfunction [3, 29, 41, 56]. Following failure of conservative intervention, or with appropriate indication, arthroscopic rotator cuff repair (RCR) tends to be the procedure of choice [15, 16, 35, 45]. An estimated 200,000–300,000 RCR procedures are performed in the United States alone each year [24, 26, 53, 54, 56], however, retear rates are high and range from 13.1% to 79% [20, 21, 27, 28, 30, 40, 56].

A variety of factors influence healing following repair and rates of retear, many have indicated poor tendon quality, tear size and patient age as possible influencing factors [21, 31, 36, 56, 64]. These conditions contribute to poor biological environment, compromising tissue healing and preventing the restoration of the biological and mechanical properties of the repaired tendon [21, 22, 23, 30, 31, 36, 56]. In response to these challenges, a variety of tissue augmentation strategies, in the form of scaffolds and patches, are being developed including periosteal patches, extracellular matrix scaffolds, allografts, autografts and xenograft of various kinds [17, 25, 56].

Scaffolds provide a footprint by which biological cells can adhere to and have been shown to encourage healing via promotion of vascularization and native tissue regeneration [30, 50, 55, 56]. Additionally, restoration of footprint dimensions and reduction of tissue strain are necessary to promote longevity of RCR. Bio‐inductive collagen implants (BCIs) made of highly porous purified type 1 collagen fibres originating from bovine tendons, cleared of any traces of animal DNA, such as the Regeneten® implant (Smith & Nephew,), have been designed with these principles in mind [7, 30].

BCIs have been applied to both full‐ and partial‐thickness RC tears (PT‐RCTs, FT‐RCTs) [6, 7, 14, 50]. BCIs can serve, in PT‐RCTs, as a means to promote tear healing and induce an accelerated rehabilitation [56]. In PT‐RCTs and FT‐RCTs that have an indication for RCR, collagen scaffold can provide an augmentation to the tendon and promote a good healing milieu [56, 57]. Additionally, its application can be useful in revision repairs and massive FT‐RCTs that may have a predisposition for failure to heal [57, 58]. The Regeneten® implant has shown promising postoperative outcomes, including decreased risk of retears, improved tendon integrity and thickness on magnetic resonance imaging (MRI), and no additional complications [14, 44]. Hence, BCIs seem to offer a promising approach for resolution of RC tears via tendon augmentation and by promoting restoration of healthy tissue.

Currently, published reviews don't include the entirety of available studies reporting on the application of BCIs to RC tears [30, 40, 55]. Thus, including all published studies on this topic addresses the current literature gap, and in doing so provides insights on current and future clinical applications on BCIs. Current findings can serve as a guide for future research and can also be a starting point for the development of more precise treatment indications, potentially leading to improved postoperative outcomes. Additionally, discussing cost‐related factors can guide informed decision‐making regarding the economic viability of BCI use in surgical practice. In light of this, the present review aims to report and discuss the outcomes of animal, histological and clinical studies exploring the application of BCIs to PT‐ and FT‐RCT treatment, in addition to reporting on cost‐related factors of this procedure. It is hypothesized that addition of a BCI to RCT management will provide functional support for tendon healing when applied to RCT treatment and that the resulting improvement of postoperative outcomes may outweigh the economic cost of the procedure. These findings could further support the on‐going use and further development of BCIs in the field of orthopaedic surgery.

## METHODS

### Eligibility criteria

Considering the authors' proficiency in various languages, studies in English, Spanish and Italian were screened. Peer‐reviewed articles of each level of evidence according to the Oxford Center for Evidence‐Based Medicine were included. Studies reporting on arthroscopic or open RCR procedures with the application of BCIs were included, studies reporting on application of BCIs without concomitant RCR were also considered for inclusion. In vitro, animal, cadaver and biomechanical studies evaluating outcomes of BCI application were considered. Any study exploring scaffolds or tissue augmentation procedures composed of anything other than purified type 1 collagen fibres originating from bovine tendons were excluded. Studies including pathologies other than PT‐ and FT‐RCTs as indication for surgery were not considered. Studies performing economic evaluation or cost‐analyses of BCI‐augmented RC procedures were included in the present review. Studies solely describing the surgical technique of BCI, technical notes, letters to editors, instructional courses, conference commentaries and reviews were not included.

### Information sources

A review of literature was performed using the Preferred Reporting Items for Systematic reviews and Meta‐Analyses (PRISMA) guidelines [39]. A systematic electronic literature search was conducted on 11 April 2024, using the CENTRAL, CINAHL, Cochrane Library, EBSCOhost, EMBASE and Google Scholar bibliographic databases. No further articles were retrieved following the initial database search.

### Search strategy

The initial search strategy was organized according to the PICO (population, intervention, comparison, outcome) structure. Any population (P) undergoing application of a BCI (I) was included in the review. When applicable, comparison between analogous intervention with and without the application of a BCI (C) was reported. Functional, histological, clinical and radiographic outcomes were extracted at final follow‐up, additionally results from economic analyses of this procedure were reported (O).

The search strategies used a combination of Medical Subject Heading (MeSH) terms and ‘title/abstract’ search. For all databases, a similar search strategy to the following was used: ‘((bioinductive) OR (collagen) OR (bioinductive collagen)) AND (implant) OR (regeneten) AND (rotator cuff))’. Differences in search key configuration were due to database configuration.

### Selection process

**Figure 1:**
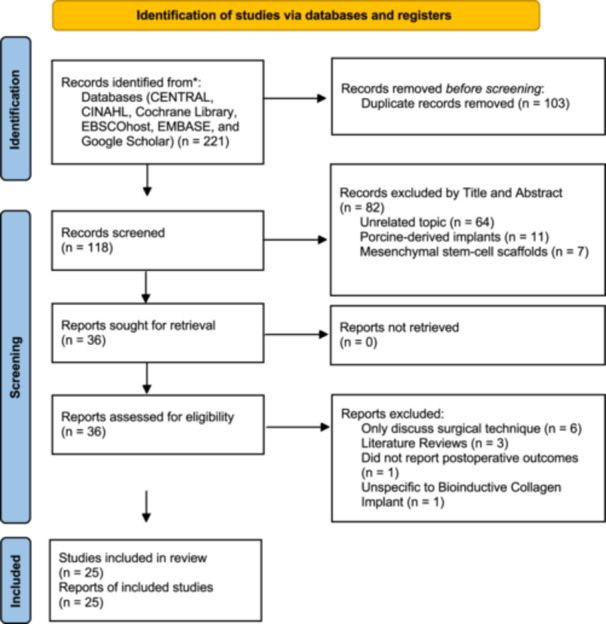
Preferred Reporting Items for Systematic reviews and Meta‐Analyses (PRISMA) flow‐chart [39.]

Screening was performed on title and abstract first, followed by full‐text appraisal by two independent reviewers (Martina Marino and Umile Giuseppe Longo) Data extraction was performed by the same reviewers. Differences, at any stage, were reconciled by mutual agreement and in case of disagreement, a third reviewer was consulted for consensus (Pieter D'Hooghe). Guidelines by Moher et al. were followed to design the PRISMA chart (Figure 1) [37].

### Data collection process

Microsoft Excel (Version 16.16.1[22101101]) was used to create charting tables onto which extracted data was recorded. Tables were formulated based on the research statement formulated using the PICO approach. Data charting was piloted by one reviewer (Martina Marino) and uncertainties in the process were discussed and resolved via consultation of the other team members. Data from all studies were charted into tables.

### Data items

General study characteristics extracted include author; year of publication; type of study; level of evidence (LoE); implant type; patient characteristics or number of animals; type of lesion when applicable; a description of the intervention, and time of follow‐up.

Data were charted onto predetermined tables, categorized based on the parameters being assessed: the first reports on human and animal histological data following BCI application (Table 1), the second reports on clinical and MRI outcomes following BCI application with or without concomitant RCR in PT‐ and FT‐RCT patients (Table S1), the third reports on the costs of utilizing BCIs for treatment of RC tears (Table 2).

**Table 1:** Human and animal histological data following BCI application.

**Reference**	**Type of study, LoE**	**Type of implant**	**Patient sex, age**	**Animal, N°**	**Original intervention**	**Comorbidities/concomitant procedures**	**Location of biopsy**	**Reason for second look**	**Time of biopsy (weeks)**	**Histology of samples**	**Mean thickness scaffold‐induced tissue (% of the underlying infraspinatus tendon, SD)**
Arnoczky et al. [2]	Case‐Report, IV	Highly porous collagen implant	M, 51		Revision FT‐RCR medium/degenerative	Hypertension/acromioplasty, biceps tenodesis	Anterolateral aspect of implant at bone attachment	Patient fell and disrupted repair	5	Presence of host cells (fibroblasts) within the interstices of the porous collagen implant; cells were aligned along the linear orientation of the collagen implant structure, and there was evidence of early collagen formation; no indication of any inflammatory or foreign body reaction within the 5‐week tissue sample.	
			F, 45		Converted high‐grade PT (Bursal) to FT‐RCR small/degenerative	None/acromioplasty, biceps tenodesis, labral chondral debridement	Antero‐lateral aspect of repair	Arthrofibrosis	8	Host incorporation throughout the implant and evidence of collagen formation on the surface and within the depths of the implant; linear orientation of new host collagen fibres along the collagen structure of the implant; no evidence of any inflammatory or foreign body reaction to the implant.	
			M, 55		Primary FT‐RCR medium/degenerative	None/acromioplasty	Multiple areas of the implant	Patient fell and disrupted repair	8		
			F, 23		Primary FT‐RCR medium/traumatic	None/acromioplasty, labral debridement	Anterior aspect of repair	Patient fell and disrupted repair	12	Increased collagen formation, maturation, and organization over the surface of the implant; remnants of the collagen implant were still present in all specimens with evidence of dissolution of the implant by invading fibroblasts; no indication of any inflammatory or foreign body reaction related to the implant in any of the tissue samples.	
			F, 43		Primary FT‐RCR large/degenerative	None/acromioplasty, biceps tenodesis, labral chondral debridement	Posterolateral aspect of repair	Pain; portion of tear not covered by implant was not healing	12		
			F, 50		Medium PT (Bursal)/degenerative	None/acromioplasty	Antero‐lateral aspect of repair	Patient's arm was jerked while walking dog on leash	12		
			F, 46		Primary FT‐RCR massive/traumatic	Glenohumneral osteoarthritis/chondroplasty, capsular release, acromioplasty	Anterior aspect of repair	Staged hemiarthroplasty	24	The newly generated tissue had the histologic appearance of a tendon (dense, regularly oriented connective tissue containing parallel rows of fibroblast within parallel bundles of collagen fibres); presence of highly oriented collagen fibres suggests functional loading of the new generated host tissue; No evidence of any remnants of the collagen implant; No evidence of any inflammatory or foreign body reaction within the tissue sample.	
Camacho‐Chacon et al. [14]	Prospecitve Case‐Series, IV	Regeneten bio‐inductive implant	1		RCR			Reoperation	24	complete healing of the lesion with a layer of new tissue on the surface of the supraspinatus tendon and its humeral footprint; new tissue adhered to the rest of the cuff and was indistinguishable from a healthy tendon;	
			29					1.5 mm biopsy punch under local anaesthesia		Newly generated tissue had the histological appearance of a tendon in all samples obtained, mature fibrous connective tissue with parallel rows of fibroblasts within parallel bundles of collagen fibres indistinguishable from the native tendon. Absence of inflammatory, scarring or ischaemic changes in all the specimens, no evidence of foreign body reaction in the tissue samples. There was no evidence of any remains of the collagen implant.	
Van Kampen et al. [59]	Experimental Animal Study, IV	Reconstituted collagen scaffolds made from highly purified, type I collagen from bovine tendons (Collagen Matrix, Inc.)		Sheep, 23	Surgical simulation of infraspinatus tendon tear, repair with implantation of collagen scaffold				6	Voids in the colla gen scaffold were completely filled with proliferating fibrovascular tissue; fibres of the collagen scaffold were clearly evident and easily distinguished from the new tissue ingrowth; no evidence of any inflammatory or foreign body reaction. (observations were consistent in all specimens)	70.8 ± 11.3 (SD)
									12	A layer of new connective tissue overlying the superior surface of the infraspinatus tendon and its bony insertion was clearly visible; New tissue was composed of fibroblasts and regularly oriented collagen fibres and was intimately attached to the underlying infraspinatus tendon; fibres of the collagen scaffold were occasionally visible in the 12‐week sections; Scaffold‐induced connective tissue demonstrated good integration into bone and there was evidence of Sharpey's fibres; no evidence of any inflammatory or foreign body reaction. (observations were consistent in all specimens)	80.5 ± 16 (SD)
									26	Layer of new connective tissue overlying the infraspinatus tendon appeared more mature and regularly‐oriented; No evidence of any remaining collagen scaffold; Bony insertion of the new tissue demonstrated evidence of a fibrocartilagenous component, suggestive of a more normal direct insertion; No evidence of any inflammatory or foreign body reaction.	91.5 ± 0.6 (SD)
									52	Histological response remained stable; Scaffold‐induced tissue was less cellular and histologically resembled tendon like (dense, regularly oriented) connective tissue and was well‐integrated with the underlying host tendon; No evidence of any inflammatory or foreign body reaction	86.1 ± 13.6 (SD)
				Sheep, 6	Surgical simulation of infraspinatus tendon tear, repair without implantation of collagen scaffold				6 and 12	In the absence of the scaffold, there was no discernible induction of new tissue over the superior surface of the in‐ fraspinatus tendon.	

Abbreviations: BCI, bio‐inductive collagen implant; F, female; M, male; PT‐ and FT‐RCTs, partial‐ and full‐thickness rotator cuff tears.

**Table 2:** Summary of reported costs of rotator cuff tear treatment with addition of BCI or general biologics.

Reference	Type of tear	Difference in costs	Difference in healed tears	Cost per healed tear (ICER)
McIntyre et al. [34]^a^	Base case	$232,468	18	$13,061
	Medium	$277,081	12	$23,550
	Large	$239,339	17	$14,188
	Massive	$62,242	41	$1525
Rognoni [42]	Type of tear	Cost or saving per healed tear (ICER)		
		NHS perspective	Societal perspective	
	Medium	27,894	–6559	
	Large	18,935	–5095	
	Massive	7926	–3295	

Abbreviations: BCI, bio‐inductive collagen implant; ICER, incremental cost‐effectiveness ratio; NHS, National Health Service.

a Results expressed per 100 treated patients.

The following parameters were extracted and included in Table 1: original intervention, comorbidities or concomitant procedures, location of biopsy, reason for second look (in humans), time of biopsy in weeks, and histology of samples, and mean thickness of scaffold‐induced tissue.

The following parameters were extracted and included in Table S1 when reported: data regarding secondary interventions, various MRI outcomes, and postoperative outcome measures including American Shoulder and Elbow Surgeons (ASES) score, ASES pain, ASES shoulder function, Brief Pain Inventory, Constant Murley Score (CMS), Constant Pain Score, Single Assessment Numeric Evaluation (SANE), Veterans RAND 12‐Item Health Survey (VR‐12) divided into mental component summary (MCS) and physical component summary (PCS), Western Ontario Rotator Cuff Index (WORC), Visual Analogue Scale for pain (VAS), simple shoulder test (SST) and range of motion (ROM) including forward flexion (FF), abduction (ABD), external rotation (ER), internal rotation (IR), forward elevation (FE). Finally, complications were reported.

Table 2 includes the following extracted parameters: type of tear, difference is costs, difference in healed tears, incremental cost‐effectiveness ratio (ICER) and mean additional procedural cost.

### Study risk of bias assessment

**Figure 2:**
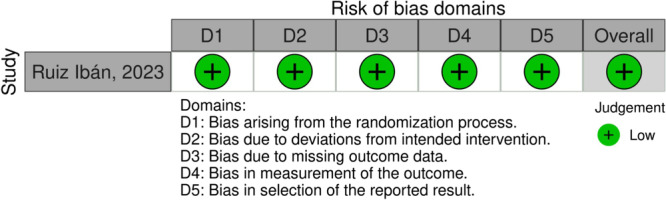
RoB2: Risk of bias assessment for randomized controlled trials [51].

**Figure 3:**
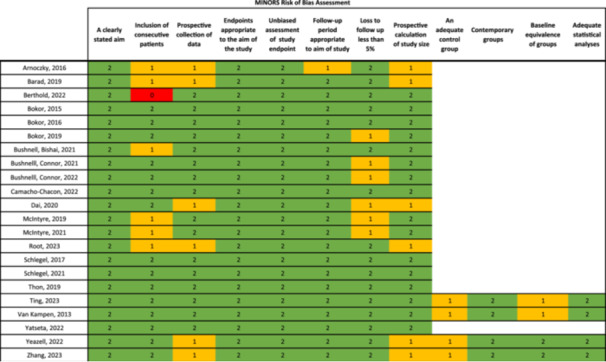
MINORS: Methodological index for nonrandomized studies [49].

Given the designs of the included studies, the risk of bias (RoB 2) tool for randomized trials [51] (Figure 2), and the methodological index for nonrandomized studies (MINORS) [49] (Figure 3) were used for risk of bias assessment.

The RoB2 tool delineates specific domains and includes ‘signalling questions’ to assess biases within randomized trials only. Each domain receives a score indicating low, moderate, serious, or critical risk of bias, contributing to an overall assessment of study quality.

The MINORS is designed to assess bias in non‐randomized studies, particularly those evaluating surgical or clinical interventions. The tool consists of 12 items, divided into two sections: methodological quality (items 1–8) and outcome reporting for cohort studies (items 9–12), each of which can be scored as either 0 (not reported), 1 (reported but inadequate), or 2 (reported and adequate). Studies can have a score ranging from 0 to 24, and the higher the overall score the better the study quality.

In the context of this review, quantifying the risk of bias of included studies helps identify concerns relevant to the available literature and provides insights into the reliability of reported study findings.

Reliability statistics were not calculated for this risk of bias assessment. To avoid discrepancies, a thorough standardized training and calibration process for all reviewers was provided along with periodic discussions to resolve any disagreements. Hence, this approach was deemed to provide a sufficient level of consistency in assessment.

### Effect measures

*p*‐Value and standard deviation values were included in tables when reported to assess statistical significance of results. A meta‐analysis was not conducted due to substantial heterogeneity in the included studies. The studies varied significantly in their methodologies, populations, interventions and outcome measures, which precluded the pooling of data. Additionally, the presence of different study designs and inconsistent reporting of results further complicated the possibility of a meaningful quantitative synthesis. Therefore, a narrative synthesis was deemed more appropriate to accurately capture and interpret the findings across the diverse body of literature. Charted data were reported in the results section according to the table it belonged to Tables 1, S1 and S2.

## RESULTS

### Study selection

Following the PRISMA protocol 25 studies were included in the present review. The PRISMA flowchart of the literature search is reported in Figure 1.

### Study characteristics

The LoE and study design of each of the included studies were: one‐level 1b randomized controlled trial (RCT); [44] one‐level 2b prospective cohort study; [58] two‐level 2b retrospective cohort studies; [61, 63] 12‐level four prospective case series [5, 6, 7, 8, 10, 11, 12, 14, 46, 47, 57, 60], three‐level four retrospective case‐series studies; [18, 32, 33] three‐level five case reports [2, 4, 43], two‐level 3b economic studies [34, 42] one‐level five experimental animal study [59].

Studies included in Table 1 [2, 14, 59] reported on human and animal histological data following BCI application, a total of 37 patients in two studies [2, 14] underwent biopsy for histological sample analysis during secondary interventions. An experimental animal study [59] compared histological evaluation of biopsy samples in 23 sheep that were subjected to BCI application versus six that did not.

Studies included in Table S1 [4, 5, 6, 7, 8, 10, 11, 12, 14, 18, 32, 33, 43, 44, 46, 47, 57, 58, 60, 61, 63] reported on clinical and MRI outcomes at follow‐up of patients that underwent application of BCI with or without RCR. One [4] study including one patient alone did not state whether the lesion was partial or full thickness, five studies [6, 7, 14, 32, 60] evaluated outcomes in both 218 FT‐ and PT‐RCT patients, seven studies [8, 10, 18, 43, 46, 47, 61] reported on 375 PT‐RCT patients, and finally eight studies [5, 11, 12, 33, 44, 57, 58, 63] reported on a total of 457 FT‐RCT patients.

Studies included in Table 2 [34, 42] report on costs of RC tear treatment in addition of BCI or general biologics.

### Risk of bias in studies

The included RCT [44] was judged as having a ‘low risk of bias’ for each of the five evaluated domains (Figure 2).

MINORS (Figure 3) [2, 4, 5, 6, 7, 8, 10, 11, 12, 14, 18, 32, 33, 43, 46, 47, 57, 58, 59, 60, 61, 63] risk of bias assessment yielded overall satisfactory results. Seven studies did not provide sufficient information to confirm consecutive patient selection. This may increase bias given that consecutive patient selection minimizes selection bias and is generally more closely representative of real‐world populations. Six included studies did not have a prospective study design and therefore scored poorly on prospective collection of data and prospective calculation of study size. Retrospective studies generally leverage existing data and in doing so can be more prone to bias such as confounding. A loss to follow‐up >5% was reported in six of the included studies. All four studies with two separate cohorts used contemporary groups and performed adequate statistical analysis. Similarly, all four studies scored inadequately for adequacy of the control group given that not all key variables that may predict outcomes had been matched for, in some instances variables were not matched validly, or the information provided was insufficient to confirm full adequacy of the control group. In two of the comparative studies, inadequate score was given for baseline equivalence of groups due to the detected differences between the control group and testing group.

### Results of individual studies

Narrative description of the results reported in Tables 1, S1 and S2 can be found in Appendix A.

### Human and animal histological data (Table 1)

Three studies reported on histological data, two of which obtained biopsy samples from humans [2, 14], one of which obtained biopsy samples from sheep [59]. All studies reported a layer of new connective tissue at 12–24‐week follow‐up, and highlighted the presence of oriented collagen fibres, suggesting functional loading. Two studies report that at 24–26‐week follow‐up, there were no identifiable remnants of the scaffold.

### Clinical and MRI outcomes following BCI application in PT‐ and FT‐RCT patients (Table S1)

#### Case reports

Two of the included studies [4, 43] are case reports, one of which explores outcomes following RCR with BCI augmentation; the other discusses BCI augmentation alone. In both cases, patients suffered from swelling and pain, followed by negative fluid culture, and identification of rice bodies, or cystic‐like structures. Resolution of inflammation occurred in both patients following secondary intervention.

#### BCI application to FT‐RCTs

Nine studies [4, 5, 7, 11, 12, 33, 44, 57, 58, 63] evaluated outcomes of BCI augmentation to treatment of FT‐RCTs.

Seven studies [5, 7, 11, 12, 33, 44, 63] reported on changes in patient‐reported outcome measures (PROMs). Three studies [7, 11, 12], two of which were consecutive [11, 12], reported statistically significant increase (*p* < 0.001) in CMS and ASES scores at 12–24 months postoperatively. Additionally, one comparative study [44] reported no significant difference in achievement of minimal clinical important difference (MCID) for ASES and CMS in FT‐RCT patients with and without BCI application. Two more studies reported improvement in ASES score at 5.2–12‐month follow‐up [5, 33], in addition to steady decrease in VAS [5, 33, 63], but no statistical significance values were reported.

Three studies [7, 44, 57] reported on tendon thickness, one [7] of which reported progressive decrease in thickness with length of follow‐up, at final follow‐up, mean thickness was reported to be 6.8 ± 0.49 mm in males and 6.2 ± 0.25 mm in females. Another study [57] reported opposite findings with progressive increase in thickness of 6.29, 6.75, 7.72 and 7.28 mm at 3, 6, 12 and 24 months postoperatively, respectively. In both cases, values remained steadily higher than published mean tendon thickness values. The third study [44] reported statistically significant differences (*p* < 0.05) in thickness of the tendon at the medial edge of the footprint and at 10 mm from it in favour of BCI implant group instead of the control group.

In one study [5], ROM values including FF, ABD and ER improved by 3°, 10° and 38°, respectively, at 6.5 ± 1.3 months follow‐up. Two studies [58, 63] compared ROM values between implant and no implant groups, one of which [63] reported statistically significant increase in ABD (*p* < 0.05), IR (*p* < 0.001) and FE (*p* < 0.001 BCI group, and *p* < 0.05 control group), nonsignificant improvement in ER in both groups. The other study [58] reported that patients in the control group had greater ER and FF at 6 weeks (*p* = 0.013; *p* = 0.044) and greater FF at 3 months (*p* = 0.020) postoperatively compared to those in the BCI group, whereas patients in the BCI group were stronger in IR and ADD at 3 months (*p* = 0.048; *p* = 0.040).

In terms of complications, comparative studies found that in the BCI group 2 patients reported minor complications, and two had serious complications, while in the control group 3 patients suffered from each, respectively; [44] patients in the control group experienced extreme pain more frequently than patients in the BCI group at 6 weeks (*p* = 0.034) and 3 months (*p* = 0.047) postoperatively, as well as greater shoulder stiffness preoperatively (*p* = 0.020), at 6 weeks (*p* < 0.001), and at 3 months (*p* = 0.004) follow‐up; [58] in both groups, there were two patients that underwent revision to reverse shoulder arthroplasty due to persistent pain and functional deficits [63].

In two consecutive studies [11, 12], incidence of retear, out of 115 patients, was reported to be 13, 19 and 21 at 3, 12 and 24 months, respectively, which caused nine reoperations. While in another study, 22 patients underwent revision surgery. In another patient cohort of 210 patients, 22 underwent revision surgery [33].

#### BCI application to PT‐RCTs

Seven studies [6, 8, 10, 18, 46, 47, 61] evaluated outcomes of BCI augmentation to treatment of PT‐RCTs.

Six studies [6, 8, 10, 18, 46, 47] reported on PROMs, all of which reported statistically significant increase in ASES score at 1.5–60‐month follow‐up. Four studies [6, 8, 46, 47], reported statistically significant improvement in CMS at 12–60‐month follow‐up.

Five studies [6, 8, 18, 46, 47] reported on defect fill‐in and tendon thickness following BCI application to PT‐RCTs. Three studies [6, 8, 18] evaluated outcomes of BCI augmentation without RCR. Two consecutive studies [6, 8] reported that in the first 12 months tendon thickness continued to increase, while at 24‐month follow‐up a slight decrease in thickness was reported, at last follow‐up two patients had new onset of a low‐grade PT‐RCT. Another study [18] found that at mean 9.9‐month follow‐up mean tendon thickness reached 6.5 ± 1.3 mm (*p* = 0.007). Three more studies evaluated outcomes following RCR and BCI augmentation [46, 47, 61]. Two consecutive studies [46, 47] reported that at 24 months MRI revealed >50% fill‐in of the defect in 90.9% of intermediate‐grade tears and 84.2% of high‐grade tears; tendon thickness was highest at 3 months in both groups, and gradually decreased at 12‐ and 24‐month follow‐ups. Nevertheless, at final 24‐month follow‐up statistically significant increase in tendon thickness from preoperative value was reported for both groups.

One comparative study [61] reported that at 3‐month follow‐up, stiffness was significantly more in the BCI group (*p* < 0.001), as opposed to patients in the control group.

#### BCI application to FT‐ and PT‐RCTs

Two studies [14, 60] did not stratify results based on partial or full‐thickness tears, while one study [32] compared outcomes of BCI application to FT‐RCTs versus PT‐RCTs.

In the first study [14], defect was filled at 6 months in 27 of 30 patients, while in the remaining patients, >50% of the tear was filled. In the second study [60], tendon integrity was achieved at 3–6 month follow‐up.

In terms of PROMs in one case, CMS and ASES scores both had statistically significant increase in value (*p* < 0.001), while VAS decreased significantly (*p* = 0.003) [14], in the other [60] postoperative improvements at follow‐up were seen for ASES score, CMS, and ROM values including FF, ABD, IR, ER.

The third study [32] compared postoperative outcomes in 90 PT‐RCT and 83 FT‐RCTs patients. In full‐thickness patients, statistically significant improvement was seen in ASES score, SANE, VR‐12 PCS, WORC and VAS at 1.5–12‐month follow‐up. In partial thickness patients, statistically significant improvement was recorded from 2 weeks up to 12 months for ASES, ASES shoulder function, SANE and VAS score, WORC, VR‐12 MCS and PCS showed improvement at 3, 6 and 12 months. Revision surgery for complications was performed in eight patients.

### Cost analysis (Table 2)

Two studies [34, 42] analysed healthcare costs with application of BCIs, both of which were based on the same clinical data sets and contextualized to the specific healthcare system being analysed. Cost‐breakdown of each can be observed in Table 2. Both studies looked at BCIs specifically and concluded that combining BCI with conventional RCR improved postoperative outcomes with only a slight cost increase, making it cost‐effective for patients.

## DISCUSSION

Reviewed literature suggests that BCI augmentation, alone and in combination with RCR, displays generally good histological, postoperative clinical and MRI outcomes in the management of both PT‐ and FT‐RCTs. From an economic standpoint, the cost‐effectiveness of the application of these scaffolds has been a matter of debate, but recent economic analyses seem to be in favour of the use of this procedure, albeit when selected and applied to appropriate patient populations.

Histological evaluation, in human and animal studies, revealed positive outcomes and has shown that there is facilitation of tendon tissue regeneration with a variety of promising histological characteristics that relate to and promote healthy RC function [2, 14, 59]. Results of evaluated studies demonstrate that BCIs consistently support rapid host‐tissue ingrowth and induce its maturation into a thick layer of regularly oriented connective tissue [59]. This organization, observed in histological sections of patients and animals with BCI augmentation, suggests functional loading of the newly generated host tissue, and thus can aid in improvement of RC function [2]. The included histological studies indicate that the implant is fully reabsorbed [2, 14]. Histological examination revealed that at 24‐month follow‐up the BCI cannot be differentiated from the surrounding native tissue. Furthermore, MRI performed 6 months postoperatively revealed that the signal from the newly formed tendon is identical to that of the original tendon [2, 14]. In summary, histological data supports the hypothesis that tissue augmentation with BCI enhances tissue quality, potentially improving the strength and longevity of RCRs across a spectrum of tear types.

In patients with full‐thickness tears, BCI augmentation was performed concomitantly to RCR in all cases; [4, 7, 11, 12, 33, 44, 57, 58, 63] only five studies [6, 8, 18, 32, 43] evaluated isolated BCI augmentation applied over PT‐RCTs. Clinical outcome measures showed a statistically significant improvement in most cases, regardless of follow‐up period. However, there were no stark differences in improvement of outcome measures in the included comparative studies [32, 44, 63]. Tendon thickness, in both PT‐ and FT‐RCTs, showed significant increase on short‐term follow‐up and was followed by a gradual reduction of thickness, however, at final 2‐year follow‐up, tendon thickness remained higher when compared to preoperative values [7, 46, 47, 55]. Given that histological studies [2, 14, 59] reveal full incorporation of the implant by 6‐month follow‐up it is likely that the decrease in thickness at 2–5 years could be due to normal remodelling of functional tissue, rather than BCI reabsorption given that this occurs early in the postoperative period [32, 44, 63].

There was a discrepancy in findings related to postoperative stiffness as a complication of BCI augmentation. One study by Yeazell et al., evaluating PT‐RCT treatment, found that stiffness was significantly higher in the BCI group as opposed to the control group [61], while another study, evaluating FT‐RCTs, instead reported that the control group experienced more stiffness preoperatively and at both follow‐up visits [58]. It has been reported that the most frequent adverse event following RCR is stiffness within 6 months, and that PT‐RCT patients are 1.5 times more likely to experience stiffness compared to their full thickness counterparts [19]. Furthermore, in a letter to editor published in response to findings by Yeazell et al., authors cite contrast with their own clinical experience and highlight lack of consideration of factors such as diabetes, preoperative stiffness and heterogeneity of surgical treatment, and delayed rehabilitation protocol as possible risk factors for development of postoperative stiffness [9]. In response, authors argue that it appeared, according to their data, that diabetes and smoking were not predominant factors affecting postoperative stiffness, however, they admit that patients were not enrolled in an accelerated rehabilitation programme [62]. This information suggests that it may not be appropriate to directly correlate postoperative stiffness as an adverse event of BCI application in PT‐RCT patients, nevertheless, due to the reported discrepancy, it is encouraged to further evaluate the possible adverse events related to BCI use.

Another interesting finding was related to two case reports following, in one case isolated BCI application and in the other application with concomitant RCR. Both patients experienced pain and swelling of the shoulder and following a secondary drainage or arthroscopic intervention revealed the presence of rice bodies [4, 43]. Both authors did not directly associate this finding with the collagen scaffold. Rice bodies are a rare phenomenon of unknown origin, and their exact pathogenesis is currently unknown, although they've been associated to conditions such as rheumatoid and tuberculous arthritis [48, 52]. Further investigation into this phenomenon in relation to BCI application would be beneficial.

In light of the generally positive outcomes associated with BCIs, their future applications are promising, for example, a recently published double‐blinded RCT [13] has suggested the use of suture‐less isolated bioinductive repair (IBR) of small to medium‐sized full‐thickness tears in patients with an intact rotator cable. Results showed that, compared to sutured repair, IBR provides sufficient structural integrity leading to robust biologic healing response [13]. This study showcases the advancements and achievements that BCIs are introducing to the field of RC treatment and the possible future trajectory of this technique.

Due to potential of collagen scaffolds to improve postoperative outcomes for RC tears and their increased use in surgical practice, concerns have been raised in terms of their cost‐effectiveness. Of the economic analysis reviewed, one concluded that the combination of BCI with RCR led to better healing rates with a marginal cost increase, making it a cost‐effective addition for patients [34]. Another stated that considering healed tears as the outcome, Regeneten® in particular seems to be a cost‐effective choice compared to the standard of care from a societal perspective [1]. These implants, inevitably bring about an additional healthcare cost, therefore their application must be carefully evaluated and likely should be reserved for specific patient populations. Nevertheless, current benefits of the procedure support its continued application in the field of orthopaedic surgery.

The limitations of the present review include the lack of statistical analysis. To include and review all the available literature on the present topic collected data were rather heterogenous, and thus statistical analysis could not be performed. Nevertheless, a review and discussion of current findings is relevant thanks to the novelty of the topic and can serve as a starting point for development of other systematic reviews, as well as high‐quality comparative studies. Additionally, there was a varied methodologic approach in data reporting, given the inclusion of cost analyses, and although reported data was not as homogeneous, the inclusion of these studies allows for a more complete understanding of the possible applications of BCIs in surgical practice. There is also inclusion of almost exclusively nonrandomized studies, hence decreasing the overall quality of reported data. Currently, only one [44] high level RCT on the present topic is available, and this suggests that an effort should be placed in producing more high‐quality data on this topic. Additionally, eligibility criteria dictated that implants had to be composed of highly porous purified type 1 collagen fibres alone, without addition of bioresorbable polymers. On the one hand, such criteria resulted in the exclusion of devices such as the CONMED BioBrace®, due to its composition including synthetic materials in addition to bovine‐derived collagen fibres. On the other hand, these parameters led to the inclusion of various studies utilizing the Regeneten® implant produced by Smith & Nephew. Despite this, studies reporting on BCIs that were not produced by Smith & Nephew, but aligning with the eligibility criteria, were eligible for inclusion in the analysis. Such protocol allowed a complete and impartial exploration of the application of BCIs to PT‐ and FT‐RCT treatment, regardless of the brand producing such devices.

## CONCLUSIONS

BCIs are a novel technology that aim to enhance the biological and mechanical properties of repaired tendon tissue by stimulating growth and healing. Overall, the findings of the present literature review align with the initial hypothesis. Several studies have shown promising results of BCI application to PT‐ and FT‐RCTs, both concomitantly and independently from RCR. Investigations report promising histological characteristics, improved clinical outcomes, increased tendon thickness, reduced defect size, and lower re‐tear rates. Nevertheless, more high‐quality RCTs are encouraged to compare the efficacy and safety of BCIs with other treatment modalities and to determine the optimal indications, techniques and implant characteristics. When considering results of cost‐analyses, the benefits of the procedure support its continued application in the field of orthopaedic surgery.

## AUTHORS CONTRIBUTIONS

**Umile Giuseppe Longo**: Conceptualization; software; validation; writing—review and editing; supervision; project administration. **Martina Marino**: Methodology; investigation; data curation; writing—original draft preparation; visualization. **Alessandro de Sire**: Writing—review and editing. **Pieter D'Hooghe**: Conceptualization; formal analysis; writing—review and editing; supervision; project administration. All authors have read and agreed to the published version of the manuscript.

## CONFLICT OF INTEREST STATEMENT

The authors have no funding to report.

## ETHICS STATEMENT

Not applicable.

## Supporting information

Supporting Information

## Data Availability

All data generated or analysed during this study are included in this published article and its Supporting Information files.

## APPENDIX A

Narrative description of results reported in Tables 1, S1 and S2.

**Human and animal histological data** (Table 1)

Three studies reported on histological data, two of which obtained biopsy samples from humans [2, 14], one of which obtained biopsy samples from sheep [59].

In the first study [2], human biopsy samples were obtained following secondary intervention due to traumatic disruption of repair (3), or other traumatic and non‐traumatic causes (4). In no cases were any inflammatory or foreign body reactions detected in response to BCI. In one patient at 5 weeks, two patients at 8 weeks and three patients at 12 weeks, host cells were aligned linearly and organized over the surface of the implant with evidence of collagen formation. At 12 weeks, remnants of the implant were still visible. In one patient, at 24 weeks, there was no evidence of BCI remnants and highly oriented collagen fibres were visualized and suggests functional loading of newly generated host tissue.

The second study [14] obtained biopsy in one patient due to necessity for reoperation at 24 weeks and 29 specimens from the remaining RCR patients at 24 weeks via a 1.5 mm biopsy punch under local anaesthesia. Histology, in the former, revealed complete healing of the lesion with a layer of new tissue that adhered to the rest of the cuff and was indistinguishable from a healthy tendon. Absence of inflammatory, scarring, or ischemic changes in all the specimens, and no evidence of foreign body reaction in the tissue samples was reported. In all cases, there was no evidence of any remains of the collagen implant. This study also reported on clinical outcomes which were charted in Table 2.

The third study [59] simulated infraspinatus tears in 23 sheep, followed by RCR and BCI application, while six animals only received RCR and served as the control group. At 6 weeks, voids in the collagen scaffold were filled with proliferating fibrovascular tissue although scaffold was still visible. By 12 weeks, a layer of new connective tissue composed of fibroblasts and regularly oriented collagen fibres was encountered, occasionally fibres of the scaffold were visible. At 26 weeks, the new tissue appeared more mature and there was no evidence of a remaining collagen scaffold. At 52 weeks, histological response had remained stable and scaffold‐induce tissue resembled that of tendon tissue. At no point was there evidence of inflammatory reaction. In the control group, there was no discernible induction of new tissue over the superior surface of the infraspinatus tendon.

**Clinical and MRI outcomes following BCI application in PT‐ and FT‐RCT patients** (Table 2).

*Case reports*

Two of the included studies [4, 43] are case reports, one of which explores outcomes following RCR with BCI augmentation; the other discusses BCI augmentation alone.

In the first case [4], at 16 weeks, there was acute massive swelling with intense pain. Complete blood count was normal and fluid aspiration showed turbid fluid with elevated white blood cell count, and rice bodies were identified. Secondary intervention included washout, debridement, placement of tubes for drainage, and IV Antibiotics. Fluid analysis, at this point, revealed negative culture. At 20 weeks from the original intervention, and at 4 weeks from the secondary intervention, swelling had subsided, and a month later, pain had almost completely disappeared.

In the second case [43], the patient had a moderate grade PT‐RCT, at 12 months from intervention, the patient reported dull aching pain and swelling. MRI investigation revealed debris in subacromial bursa with small bodies of intermediate density and cystic‐like structures eroding through deltoid fascia and into the inferior acromion, resulting in local soft‐tissue and osseous destruction. At this time, arthroscopic subacromial debridement was performed, BCI was mostly incorporated, and rice bodies were identified. Fluid culture was negative. At 24 months from original intervention, and at 12 months from secondary intervention, the patient had good recovery and was able to return to professional tennis.

*BCI application to FT‐RCTs*

Nine studies [4, 5, 7, 11, 12, 33, 44, 57, 58, 63] evaluated outcomes of BCI augmentation to treatment of FT‐RCTs.

In the first study [5], five male patients received RCR and BCI augmentation. Outcome measures were recorded preoperatively and at 6.5 ± 1.3 months postoperatively, all showed improvement. ASES score improved by 56.3 points, SANE score improved by 78.25 points, VAS score decreased by 5.9 points, and FF, ABD, ER improved by 3°, 10° and 38° respectively.

The second study [7] reported MRI and outcome scores preoperatively and at 3, 6, 12 and 24 months postoperatively, in nine FT‐RCT patients undergoing BCI augmented RCR. Tendon thickness decreased with length of follow‐up and at final follow‐up mean thickness was reported to be 6.8 ± 0.49 mm in males and 6.2 ± 0.25 mm in females, however, thickness remained steadily higher than published mean tendon thickness values. Statistically significant increase (*p *< 0.001) in CMS and ASES scores was recorded from preoperative value to 24 months postoperatively.

Two consecutive studies [11, 12] evaluated postoperative outcomes following RCR and BCI application for medium and large FT‐RCTs. one hundred and fifteen patients were included in the follow‐up period. Incidence of retear at 3, 12 and 24 months was 13, 19 and 21, respectively. Nine reoperations were performed. Statistically significant (*p* > 0.001) increase in ASES score and CMS and decrease in ASES pain were found at all follow‐up intervals for both large and massive tears. ASES shoulder function had a statistically significant increase in score at 12 and 24 months only.

The fifth study [33] evaluated outcomes of RCR with BCI augmentation in 210 small, medium, large and massive FT‐RCT patients. Outcomes were reported at baseline, 6, and 12 months. Increase in ASES pain, ASES shoulder function, SANE, VR‐12 MCS and PCS, and WORC was seen at both follow‐up times. VAS also decreased steadily. Statistical significance was not reported. Twenty‐two patients underwent revision surgery.

The sixth study [57] reported tendon thickness following RCR and BCI placement in 11 patients with large and 12 with massive FT‐RCTs and found a progressive increase in this value. Thickness measured via ultrasound was 6.29, 6.75, 7.72 and 7.28 mm at 3, 6, 12 and 24 months postoperatively, respectively, statistical significance was not reported.

The seventh study by Ruiz Ibán et al. [44] is an RCT evaluating RCR with (61 patients) and without (63 patients) BCI application for treatment of FT‐RCTs. There were statically significant fewer retears in the BCI group compared to the control group (*p* = 0.0106; OR = 0.261). Statistically significant differences (*p* < 0.05) in thickness of the tendon at the medial edge of the footprint, and 10 mm medial to it were recorded, at 12 months, in favour of BCI implant group, while no statistically significant difference was found at 20 mm medial measurement. In terms of ASES and CMS at 12 months, 75% and 76.7% of patients in the BCI group achieved MCID as opposed to 80% and 81.7% in those who did not receive the implant. In the BCI group 2, patients reported minor complications, and two had serious complications, while in the control group, 3 patients suffered from each, respectively.

The eighth study [58] compared application of BCI implant versus no implant during revision RCRs. Patients were matched for worker's compensation, age, and tear size. The BCI group included 19 patients and the control group included 32. Only statistically significant differences were reported. Patients in the control group experienced extreme pain more frequently than patients in the BCI group at 6 weeks (*p* = 0.034) and 3 months (*p* = 0.047) postoperatively, as well as greater shoulder stiffness preoperatively (*p* = 0.020), at 6 weeks (*p* < 0.001), and at 3 months (*p* = 0.004) follow‐up. Patients in the control group had greater ER and FF at 6 weeks (*p* = 0.013; *p* = 0.044) and greater FF at 3 months (*p *= 0.020) postoperatively compared to those in the BCI group. Patients in the control group were stronger in ABD than patients in the BCI group preoperatively (*p *= 0.028), whereas patients in the BCI group were stronger in IR and ADD at 3 months (*p* = 0.048; *p *= 0.040).

Finally, Zhang et al. [63] also compared patients undergoing RCR due to large or massive FT‐RCTs with and without BCI application. Both groups were composed of 24 patients and outcomes were reported preoperatively and at follow‐up. Statistically significant (*p* < 0.001) improvement in VAS and SST was found in both groups. In terms of ROM, statistically significant increase in ABD (*p* < 0.05), IR (*p* < 0.001) and FE (*p* < 0.001 BCI group, and *p* < 0.05 control group) was reported. ER improved in both groups but changes were not statistically significant. In both groups, there were two patients that underwent revision to reverse shoulder arthroplasty due to persistent pain and functional deficits.

*BCI application to PT‐RCTs*

Seven studies [6, 8, 10, 18, 46, 47, 61] evaluated outcomes of BCI augmentation to treatment of PT‐RCTs.

Two consecutive studies [6, 8] evaluated MRI and postoperative outcome measures following subacromial bursectomy and decompression with addition of BCI. At baseline, four patients suffered from intermediate grade and six from high‐grade PT‐RCTs, by 12‐month MRI follow up no tear was visible in seven patients, while three had low‐grade PT‐RCTs. In the first 12 months tendon thickness continued to increase, and by 24 months a slight decrease in thickness was reported. At 60 months in eight patients, no negative change in quality was reported. In the remaining two patients there was an onset of new low‐grade PT‐RCT. Statistically significant improvement in ASES score, ASES pain, CMS, and Constant pain score were reported at 24 and 60 months.

The third study [10] reported patients with grades 1, 2 and 3 PT‐RCTs, 31 patients underwent concomitant tear completion RCR and 241 underwent isolated bio inductive repair. Data were not stratified based on this parameter. Statistically significant improvement in ASES pain, SANE and VR‐12 PCS were found at 0.5, 1.5, 3, 6 and 12 months. Statistically significant improvement in ASES score and WORC was recorded at 1.5, 3, 6 and 12 month follow‐up. ASES shoulder function had statistically significant improvement at 0.5, 3, 6 and 12 months. Finally, VR‐12 MCS only had statistically significant improvement at 12 months.

The fourth study [18] reported on 24 PT‐RCT patients that underwent isolated BCI augmentation. At mean follow‐up of 9.9 months mean tendon thickness reached 6.5 ± 1.3 mm (*p* = 0.007). Additional statistically significant improvement of ASES score and VAS were reported (*p* < 0.001). One traumatic re‐tear occurred during the follow‐up period.

Two more consecutive studies [46, 47] evaluated postoperative outcomes following RCR with BCI in high and intermediate‐grade PT‐RCTs. At 24 months, MRI revealed >50% fill‐in of the defect in 90.9% of intermediate‐grade tears and 84.2% of high‐grade tears. Tendon thickness was highest at 3 months in both groups, but gradually decreased at 12‐ and 24‐month follow‐ups. Nevertheless, at final 24‐month follow‐up statistically significant increase in tendon thickness from preoperative value was reported for both groups (*p* = 0.012 for intermediate grade, and *p* = 0.003 for high‐grade tears). In both groups, ASES increased significantly at 3 and 12 months, while CMS increased significantly in high‐grade tears at 3 months and in both groups at 12 months. Eight complications were recorded, but none were reported to be associated with BCI.

Finally, the last study [61] compared stiffness in PT‐RCT patients that had undergone RCR augmented with BCI and in those that only underwent repair. Each group was composed of 32 patients. Stiffness, at 3‐month follow‐up, was significantly more in the BCI group (*p* < 0.001), with eight patients from the group experiencing stiffness as opposed to just one in the control group.

*BCI application to FT‐ and PT‐RCTs*

Two studies [14, 60] did not stratify results based on partial or full‐thickness tears, while one study [32] compared outcomes of BCI application to FT‐ versus PT‐RCTs.

The first study [14] performed BCI augmentation alone in PT‐RCTs and BCI augmentation with RCR in FT‐RCTs, and included 30 patients. In 27 patients defect was filled at 6 months, while in the remaining patients >50% of the tear was filled. At 12 months none of the patients showed degeneration or variation in thickness, and neo‐tendon was indistinguishable from original tissue. CMS and ASES score both had statistically significant increase in value (*p* < 0.001). While VAS decreased significantly (*p* = 0.003).

The second study [60] reported outcomes of RCR and BCI augmentation in six myotendinous junction PT‐RCT and FT‐RCT patients. Tendon integrity was achieved at 3–6 month follow‐up. Postoperative improvements at follow‐up were seen for ASES score, CMS and ROM values including FF, ABD, IR and ER. Statistical significance was not reported.

The third study [32] compared postoperative outcomes in 90 PT‐RCT and 83 FT‐RCTs patients. The former only received BCI application, and the latter underwent RCR with BCI augmentation. In full thickness patients, statistically significant improvement was seen in ASES score, SANE, VR‐12 PCS, WORC, and VAS starting from 1.5 months, and at 3‐, 6‐, and 12‐month follow up, while ASES shoulder function significantly improved at all follow‐ups, excluding that performed at 1.5 months. There was no statistically significant improvement of VR‐12 MCS at any point. In partial thickness patients, statistically significant improvement was recorded from 2 to 12 months, at each follow‐up interval, for ASES shoulder function, SANE, and VAS score. WORC displayed improvement at 1.5, 3, 6 and 12 months, while VR‐12 MCS and PCS showed improvement at 3, 6 and 12 months. Finally, ASES score significantly improved at 2 weeks, 3, 6 and 12 months, but not at 1.5 months. Revision surgery for complications was performed in eight patients.

*Cost analysis* (Table 2)

Three studies [34, 38, 42] analysed healthcare costs with application of BCIs, two of which [34, 42] were based of the same clinical data sets and contextualized to the specific healthcare systems being analysed.

The first study [34] investigated whether the use of BCI‐augmented RCR was cost‐effective when compared to RCR alone in the treatment of FT‐RCTs. Combination of BCI and RCR was found to result in an incremental cost of $232,468 and an additional 18 healed RC tears per 100 treated patients over 1 year. The estimated incremental cost‐effectiveness ratio (ICER) was $13,061 per healed RC tear compared to conventional RCR alone. When return to work was included in the model, BCI in addition to RCR was found to be cost‐saving, cost‐effectiveness improved with tear size with the largest benefit seen in massive tears compared to large tears, as well as patients at higher risk of retearing. This analysis concluded that combining BCI with conventional RCR led to better healing rates with only a slight cost increase, making it cost‐effective for patients.

The second study [38] aimed to identify intraoperative drivers of cost associated with RCR through data retrieval via an institutional database. The procedural level factor associated with the highest charges in the analysis was the use of biologics, adding $17,791 (*p* < 0.001). Additionally, procedural level factors were found to be the most significant drivers of operative cost in arthroscopic RCRs.

The third study [42] assessed the cost‐effectiveness of the Regeneten® implant combined with standard of care (SOC), in this case being the surgical repair of the RC, compared to SOC alone from both National Healthcare Service (NHS) and societal perspectives in Italy. Over a 1‐year period, healed lesions were 90.70% and 72.90% for surgical repair of RC tears with and without Regeneten®, respectively. Considering the NHS perspective, mean costs per patient were €7828 and €4650 for the two strategies, respectively, leading to an ICER of €17,857 per healed tear. From the societal perspective, whose analysis included patients' productivity losses, which were estimated based on the weeks missed for the return to work after surgery, the mean costs per patient were €12,659 for SOC and €11,784 for Regeneten®, thus showing savings of €4918 per healed tear when BCI is used. Considering healed tears as the outcome, our findings showed the cost‐effectiveness profile of Regeneten® and that it may be a cost‐saving choice compared to SOC from the societal perspective.
